# Cardiometabolic Risk Factors Among 1.3 Million Adults With Overweight or Obesity, but Not Diabetes, in 10 Geographically Diverse Regions of the United States, 2012–2013

**DOI:** 10.5888/pcd14.160438

**Published:** 2017-03-09

**Authors:** Gregory A. Nichols, Michael Horberg, Corinna Koebnick, Deborah Rohm Young, Beth Waitzfelder, Nancy E. Sherwood, Matthew F. Daley, Assiamira Ferrara

**Affiliations:** 1 Kaiser Permanente Northwest, Portland, Oregon; 2 Kaiser Permanente Mid-Atlantic States, Rockville, Maryland; 3 Kaiser Permanente Southern California, Pasadena, California; 4 Kaiser Permanente Hawaii, Honolulu, Hawaii; 5 HealthPartners Institute, Bloomington, Minnesota; 6 Kaiser Permanente Colorado, Denver, Colorado; 7 Kaiser Permanente Northern California, Oakland, California

## Abstract

**Introduction:**

Various phenotypes of overweight and obesity pose various health risks. The objective of this study was to determine the prevalence of 4 commonly measured cardiometabolic risk factors (CRFs) among adults with overweight or obesity, but not diabetes, at the time of the study.

**Methods:**

We analyzed data for 1,294,174 adults (aged ≥20 y) who were members of one of 4 integrated health systems. Each cohort member had a body mass index in 2012 or 2013 that indicated overweight or obesity. We determined the presence of 4 CRFs within 1 year of the first BMI measurement: elevated blood pressure (systolic ≥130 mm Hg or diastolic >85 mm Hg or ICD-9-CM [*International Classification of Diseases, Ninth Revision, Clinical Modification*] diagnosis code 401.0–405.9); elevated triglycerides (≥150 mg/dL or ICD-9-CM 272.1); low high-density lipoprotein cholesterol (<40 mg/dL for men or <50 mg/dL for women or ICD-9-CM 272.5); and prediabetes (fasting glucose 100–125 mg/dL or HbA_1c_ 5.7%–6.4% or ICD-9-CM 790.2x). We tested the risk of having 1 or more CRFs after adjusting for obesity class and demographic characteristics with multivariable logistic regression.

**Results:**

Among participants with overweight (52.5% of the sample), 18.6% had none of the 4 CRFs. Among the 47.5% of participants with obesity, 9.6% had none; among participants with morbid obesity, 5.8% had none. Age was strongly associated with CRFs in multivariable analysis.

**Conclusion:**

Almost 10% of participants with obesity had no CRFs. Overweight or obesity increases cardiometabolic risk, but the number and type of CRFs varied substantially by age, even among participants with morbid obesity.

## Introduction

More than two-thirds of US adults have a body mass index (BMI) that indicates overweight or obesity (1), yet the prevalence of diabetes is approximately 9% (2), and the estimated lifetime risk of diabetes (40%) is lower than the prevalence of overweight or obesity (3). Thus, although obesity is a major risk factor for type 2 diabetes, the condition does not develop in many individuals with excess weight. A study based on the National Health and Nutrition Examination Survey (NHANES) for 1999 through 2004, which included individuals with diabetes, showed that 51% of adults with overweight and 32% of adults with obesity had no cardiometabolic risk factors (CRFs) or only one (4), which suggests that various phenotypes of overweight and obesity may pose various health risks. Because diabetes is a consequence of CRFs, preventive efforts should be implemented before diabetes develops. Characterization of individuals with overweight or obesity, but not diabetes, is needed. The ability of health systems to identify phenotypes of overweight or obesity through the use of electronic health records (EHRs) would facilitate efficient allocation of preventive resources to reduce the incidence of diabetes and cardiovascular disease (CVD). The objective of our study was to determine the proportion of adults whose BMI indicated overweight or obesity and who did not have diabetes but did have 4 commonly measured CRFs (elevated blood pressure, elevated triglycerides, low high-density lipoprotein [HDL] cholesterol, and prediabetes) in a large diverse cohort in a consortium of 4 health care delivery systems.

## Methods

Patient Outcomes Research to Advance Learning (PORTAL) is a consortium of 4 health care delivery systems — Kaiser Permanente (Colorado, Georgia, Hawaii, Mid-Atlantic States, Northern California, Northwest, and Southern California), GroupHealth Cooperative (Washington State), HealthPartners (Minnesota), and Denver Health — and their 10 research centers (5). Together, these health care delivery systems serve 12 million individuals in 11 states and the District of Columbia. PORTAL was one of 11 clinical data research networks (CDRNs) funded by the Patient-Centered Outcomes Research Institute (6). Each CDRN was required to create a “weight cohort” of individuals in its network. The development and analysis of the PORTAL cohort were approved by the Kaiser Permanente Southern California Institutional Review Board; the other sites ceded review to this board.

### Study sample

The PORTAL weight cohort is described elsewhere (7). Briefly, the cohort consisted of adults (aged ≥18 y) who were members on December 31, 2013; each member had at least 12 months of continuous membership at some point between January 1, 2012, and December 31, 2013, and each had a primary care encounter in 2012 or 2013, during which a weight was recorded. We excluded members whose EHRs lacked data on a height measurement and women who were pregnant in 2012 or 2013. The PORTAL weight cohort consisted only of adults with a body mass index (BMI, weight in kilograms [kg] divided by height in meters [m] squared) of 23.0 or greater, so the full range of data on adults with normal weight (BMI, 18.5−24.9) or underweight (BMI <18.5) were not available. We excluded adults aged 18 or 19 to make our data comparable with data from NHANES. We also excluded adults who had any indication of diabetes in their EHR, regardless of diagnosis, using the SUPREME-DM (SUrveillance, PREvention, and ManagEment of Diabetes Mellitus) algorithm; this algorithm requires 1 inpatient diagnosis (250.x) from the *International Classification of Diseases, Ninth Revision, Clinical Modification* (ICD-9-CM) or any combination of 2 outpatient events (diagnoses, diagnostic laboratory tests, or fulfillment of anti-hyperglycemic medication) (8).

These exclusions resulted in a cohort of 3,434,066 adults. We further excluded adults who lacked complete data on the 4 CRFs of interest, resulting in an analytic sample of 1,294,174. To assess the effect of missing data, we compared the demographic characteristics of adults with complete CRF data and adults without complete CRF data and conducted sensitivity analyses by repeating our primary analyses among the total cohort, assuming adults with a missing CRF value did not have that risk factor.

### Classification of overweight and obesity

We used the first available record of weight in 2012 or 2013 as the index date and calculated BMI from the nearest available height as the index value and classified the adult as overweight (BMI of 23.0–27.4 if Asian or 25.0–29.9 otherwise) or obese (BMI ≥27.5 if Asian or ≥30.0 otherwise) (9). Our large cohort allowed us to further categorize adults with obesity: obese (Class I, BMI 27.5–34.9 if Asian or 30.0–34.9 otherwise); severely obese (Class II, BMI 35.0–39.9); or morbidly obese (Class III, BMI ≥40.0).

### Cardiometabolic risk factors

Blood pressure and laboratory values (triglycerides, HDL cholesterol, fasting plasma glucose, and glycated hemoglobin [HbA_1c_]) were collected from the EHR by using the value closest to and up to 1 year before the index date. We defined 4 dichotomized (yes/no) CRFs: 1) elevated blood pressure (systolic value of ≥130 mm Hg or diastolic value of >85 mm Hg or an ICD-9-CM code from 401.0 to 405.9); 2) elevated triglycerides (≥150 mg/dL or an ICD-9-CM code of 272.1); 3) low HDL cholesterol (<40 mg/dL for men and <50 mg/dL for women or an ICD-9-CM code of 272.5); and 4) prediabetes (HbA_1c_ value of 5.7%–6.4% or a fasting glucose value of 100–125 mg/dL or an ICD-9-CM code of 790.2x). We included ICD-9-CM codes for each CRF to allow for treated adults to be assigned appropriately to a category of elevated risk, regardless of clinical values.

### Statistical analyses

We first tabulated data on our cohort descriptively. Because of the cohort’s large size, we chose not to tabulate *P* values because even small clinically insignificant differences were statistically significant at a *P* value <.001 (1-way analysis of variance). We stratified the data by age group to demonstrate the importance of age to the type and number of CRFs; we constructed a multiple logistic regression model using SAS version 9.4 (SAS Institute, Cary, North Carolina) to assess the independent contribution of demographic characteristics and overweight or obesity to the probability of having at least 1 CRF.

## Results

Of the 1,294,174 adults included in the study, 52.5% were overweight and 47.5% were obese (Table 1). Adults with obesity were about 3 years younger and more likely to be women and non-Hispanic black or Hispanic than adults with overweight. In each successive obesity class (Class I through Class III), we observed a younger age and a higher proportion of women and non-Hispanic black adults. Crude and age-adjusted mean and median values of the CRFs were generally similar among adults with overweight and adults with obesity across obesity classes; age adjustment slightly magnified the differences, particularly for blood pressure and triglycerides.

**Table 1:** Characteristics of a Sample of Adults (Aged ≥20 y) With Overweight or Obesity, but Not Diabetes, by Overweight or Obesity Class, the PORTAL Weight Cohort, 2012–2013^a^^,^ b

Characteristic	Total	Overweight (BMI 25.0–29.9)^c^	Obese (BMI ≥30.0)	Obesity Class		
				Class I (BMI 30.0–34.9)^d^	Class II (BMI 35.0–39.9)	Class III (BMI ≥40.0)
**No. (%)**	1,294,174 (100)	679,034 (52.5)	615,140 (47.5)	371,939 (28.7)	149,517 (11.6)	93,684 (7.2)
**Mean age (SD), y**	54.0 (15.0)	55.5 (15.3)	52.4 (14.5)	53.7 (14.5)	51.5 (14.3)	48.4 (14.0)
**Age group, %**						
20–34 y	11.3	10.2	12.5	10.6	13.5	18.4
35–49 y	26.5	24.3	29.0	27.4	30.0	33.7
50–64 y	36.4	35.9	36.9	37.6	36.9	34.2
65–79 y	21.7	24.1	19.0	21.1	17.6	12.8
≥80 y	4.2	5.6	2.6	3.3	1.9	0.9
**Sex**						
Male	49.7	53.6	45.4	50.3	41.1	32.6
Female	50.3	46.4	54.6	49.7	58.9	67.4
**Race/ethnicity**						
Non-Hispanic white	49.2	49.2	48.9	49.1	49.1	47.7
Non-Hispanic black	11.2	8.8	13.9	12.0	15.0	19.5
Hispanic	24.5	22.6	26.6	26.9	26.7	25.1
Asian	9.2	13.1	4.8	6.1	3.5	2.0
Hawaiian/Pacific Islander	0.9	1.0	0.9	0.8	0.9	1.1
American Indian/Alaska Native	0.5	0.4	0.6	0.5	0.6	0.7
**Systolic blood pressure, mm Hg**						
Unadjusted mean (SD)	126 (15)	125 (15)	128 (15)	127 (15)	128 (14)	130 (15)
Age-adjusted mean (SE)	116 (0.05)	111 (0.07)	119 (0.07)	116 (0.09)	119 (0.14)	123 (0.17)
Median (IQR)	126 (19)	124 (20)	128 (18)	126 (19)	128 (17)	130 (18)
**Diastolic blood pressure, mm Hg**						
Unadjusted mean (SD)	74 (10)	73 (10)	76 (10)	75 (10)	76 (10)	77 (11)
Age-adjusted mean (SE)	78 (0.03)	75 (0.05)	79 (0.05)	79 (0.06)	80 (0.10)	81 (0.12)
Median (IQR)	74 (13)	73 (13)	76 (13)	75 (13)	76 (12)	77 (13)
**Triglycerides, mg/dL**						
Unadjusted mean (SD)	137 (81)	130 (78)	144 (83)	145 (85)	147 (83)	139 (75)
Age-adjusted mean (SE)	141 (0.27)	128 (0.36)	150 (0.40)	153 (0.54)	152 (0.80)	139 (0.88)
Median (IQR)	117 (82)	110 (77)	125 (85)	124 (87)	127 (86)	122 (78)
**High-density lipoprotein cholesterol, mg/dL**						
Unadjusted mean (SD)	51 (13)	53 (14)	49 (13)	49 (13)	48 (12)	48 (12)
Age-adjusted mean (SE)	44 (0.04)	48 (0.06)	42 (0.06)	43 (0.08)	41 (0.12)	39 (0.14)
Median (IQR)	49 (17)	50 (17)	47 (16)	47 (16)	46 (15)	46 (15)
**Fasting glucose, mg/dL^e^**						
Unadjusted mean (SD)	95 (11)	95 (11)	96 (12)	96 (11)	96 (12)	97 (12)
Age-adjusted mean (SE)	86 (0.04)	82 (0.05)	86 (0.06)	86 (0.07)	86 (0.12)	87 (0.16)
Median (IQR)	94 (13)	94 (12)	95 (13)	95 (13)	95 (14)	95 (15)
**HbA_1c_ e**						
Unadjusted mean (SD)	5.6% (1.0)	5.5% (1.0)	5.6% (1.1)	5.6% (1.1)	5.6% (1.1)	5.6% (1.1)
Age-adjusted mean (SE)	5.4% (0.01)	5.3% (0.01)	5.4% (0.01)	5.4% (0.01)	5.4% (0.02)	5.5% (0.02)
Median (IQR)	5.7% (0.4)	5.7% (0.4)	5.8% (0.5)	5.7% (0.5)	5.8% (0.5)	5.8% (0.4)

Abbreviations: BMI, body mass index (kg/m^2^); HbA_1c_, glycated hemoglobin; IQR, interquartile range; PORTAL, Patient Outcomes Research to Advance Learning; SD, standard deviation; SE standard error.

a Data source: PORTAL weight cohort (7).

b All between-group statistical comparisons are significant at *P* < .001 (1-way analysis of variance) because of the large sample size.

c Overweight defined as BMI of 23.0–27.4 for Asians and 25.0–29.9 for all other race/ethnicities.

d Class 1 obesity defined as BMI of 27.5–34.9 for Asians and 30.0–34.9 for all other race/ethnicities.

e To be included in the data set, the electronic health record for an individual was required to have a value for either a fasting glucose test (84.7% of the total) or an HbA_1c_ test (40.4%).

Of the 4 CRFs studied, elevated blood pressure was the most prevalent (59.9%), followed by prediabetes (47.2%), low HDL cholesterol (33.7%), and elevated triglycerides (32.2%) (Table 2). The prevalence of these CRFs increased across overweight and obesity classes, except for triglycerides, where prevalence for obesity Class III was lower than for obesity Classes I and II. The prevalence of each CRF varied widely among age groups.

**Table 2:** Proportion of Adults (Aged ≥20 y) With Overweight or Obesity, but Not Diabetes, by Cardiometabolic Risk Factor, Age Group, and Weight Class, the PORTAL Weight Cohort, 2012–2013^a^^,^ b ^,^ c

Risk Factor, by Age Group	Total	Overweight (BMI 25.0–29.9)^d^	Obese (BMI ≥30.0)	Obesity Class		
				Class I (BMI 30.0–34.9)^e^	Class II (BMI 35.0–39.9)	Class III (BMI ≥40.0)
**Elevated blood pressure^f^**						
20–34 y	31.7	24.3	38.3	32.9	39.3	49.5
35–49 y	45.0	36.2	53.1	47.7	55.9	66.2
50–64 y	63.8	56.8	71.4	67.6	74.9	81.9
65–79 y	80.3	76.5	85.6	83.7	88.2	92.0
≥80 y	90.6	89.5	93.1	92.6	94.4	95.6
All	59.9	55.0	65.2	62.6	67.1	72.1
**Elevated triglycerides^g^**						
20–34 y	26.6	20.7	31.9	30.9	33.9	31.7
35–49 y	34.7	30.7	38.3	39.0	39.2	34.8
50–64 y	34.2	31.0	37.8	38.1	39.0	34.4
65–79	30.3	27.3	34.6	34.3	36.0	33.7
≥80 y	24.7	22.7	29.2	28.7	30.3	31.9
All	32.2	28.5	36.4	36.5	37.7	33.9
**Low high-density lipoprotein cholesterol^h^**						
20–34 y	41.1	30.0	51.0	43.9	55.1	62.5
35–49 y	39.1	31.4	46.2	42.4	49.5	53.9
50–64 y	31.0	25.0	37.5	34.8	40.5	44.0
65–79 y	28.4	24.2	34.5	32.5	37.5	40.4
≥80 y	29.6	27.3	35.0	33.9	38.2	39.4
All	33.7	27.0	41.1	37.3	44.6	50.2
**Prediabetes^i^**						
20–34 y	22.1	16.2	27.5	22.6	28.7	37.1
35–49 y	38.2	32.1	43.8	40.3	46.0	51.7
50–64 y	52.1	47.3	57.3	54.9	59.7	63.6
65–79 y	59.9	56.1	65.2	63.7	67.4	70.8
≥80 y	63.1	61.3	67.5	66.8	69.9	69.2
All	47.2	43.3	51.4	49.7	53.0	55.7

Abbreviations: BMI, body mass index (kg/m^2^); HbA_1c_, glycated hemoglobin; ICD-9-CM, *International Classification of Diseases, Ninth Revision, Clinical Modification*; PORTAL, Patient Outcomes Research to Advance Learning.

a Data source: PORTAL weight cohort (7).

b All values are percentages.

c All between-group comparisons are significant at *P* < .001 (1-way analysis of variance) because of the large sample size.

d Overweight defined as BMI of 23.0–27.4 for Asians and 25.0–29.9 for all other race/ethnicities.

e Class 1 obesity defined as BMI of 27.5–34.9 for Asians and 30.0–34.9 for all other race/ethnicities.

f Defined as systolic value of ≥130 mm Hg or diastolic value of >85 mm Hg or ICD-9-CM code from 401.0 to 405.9.

g Defined as ≥150 mg/dL or an ICD-9-CM code of 272.1.

h Defined as <40 mg/dL for men and <50 mg/dL for women or an ICD-9-CM code of 272.5.

i Defined as HbA_1c_ value of 5.7%–6.4% or a fasting glucose value of 100–125 mg/dL or an ICD-9-CM code of 790.2x). To be included in the data set, the electronic health record for an individual was required to have a value for either a fasting glucose test (84.7% of the total) or an HbA_1c_ test (40.4%).

The prevalence of having none of the CRFs was 14.3% and varied by degree of overweight or obesity and age (Table 3). Among adults with overweight (52.5% of the sample), 18.6% had none of the 4 CRFs. Among the 47.5% adults with obesity, 9.6% of had none of the CRFs, and among adults with morbid obesity, 5.8% had none. Prevalence of having none of the CRFs was 29.2% among adults aged 20 to 34 but ranged in this age group from 40.0% (overweight) to 10.4% (obesity Class III). Only 6.3% of adults aged 65 to 79 had no CRFs; prevalence in this age group ranged from 8.4% (overweight) to 1.4% (obesity Class III).

**Table 3:** Proportion of Adults (Aged ≥20 y) With Overweight or Obesity, but Not Diabetes, by Number of Cardiometabolic Risk Factors^a^, Age Group, and Weight Class, the PORTAL Weight Cohort, 2012–2013^b^^,^ c ^,^ d

No. of Risk Factors, by Age Group	Total	Overweight (BMI 25.0–29.9)^e^	Obese (BMI ≥30.0)	Obesity Class		
				Class I (BMI 30.0–34.9)^f^	Class II (BMI 35.0–39.9)	Class III (BMI ≥40.0)
**None**						
20–34 y	29.2	40.0	19.5	25.0	16.4	10.4
35–49 y	19.3	26.4	12.7	15.4	10.7	6.9
50–64 y	12.1	16.4	7.5	9.0	5.9	3.9
65–79 y	6.3	8.4	3.4	4.0	2.6	1.4
≥80 y	2.8	3.5	1.4	1.6	0.9	0.8
All	14.3	18.6	9.6	11.2	8.1	5.8
**1 Risk factor**						
20–34 y	35.0	36.2	33.9	35.7	33.7	30.3
35–49 y	31.0	34.1	28.1	30.0	26.7	24.2
50–64 y	28.8	32.6	24.8	26.4	23.1	20.6
65–79 y	26.6	30.1	21.7	23.2	19.3	17.6
≥80 y	24.9	26.8	20.3	20.8	18.6	18.2
All	29.5	32.4	26.2	27.5	24.8	23.2
**2 Risk factors**						
20–34 y	23.3	17.4	28.5	25.5	30.2	33.2
35–49 y	28.3	25.0	31.4	30.2	32.2	33.9
50–64 y	32.2	30.5	34.0	33.6	33.8	35.9
65–79 y	37.4	37.1	37.8	37.9	37.5	37.8
≥80 y	42.2	42.4	41.8	42.3	40.5	37.5
All	31.7	30.1	33.5	33.0	33.6	35.0
**3 Risk factors**						
20–34 y	10.3	5.5	14.6	11.5	15.8	20.3
35–49 y	16.4	11.7	20.7	18.6	22.2	25.4
50–64 y	19.6	15.7	23.7	22.2	25.4	27.1
65–79 y	21.2	18.0	25.6	24.5	27.6	28.9
≥80 y	21.7	20.1	25.3	24.6	27.0	31.2
All	18.1	14.5	22.1	20.6	23.6	25.5
**4 Risk factors**						
20–34 y	2.2	0.9	3.5	2.3	3.9	5.8
35–49 y	5.0	2.8	7.1	5.8	8.2	9.6
50–64 y	7.3	4.8	10.0	8.8	11.8	12.5
65–79 y	8.5	6.4	11.4	10.4	13.0	14.3
≥80 y	8.4	7.2	11.2	10.7	13.0	12.3
All	6.4	4.4	8.7	7.7	9.9	10.6

Abbreviations: BMI, body mass index (kg/m^2^); HbA_1c_, glycated hemoglobin; HDL, high-density lipoprotein; ICD-9-CM, *International Classification of Diseases, Ninth Revision, Clinical Modification*; PORTAL, Patient Outcomes Research to Advance Learning.

a We defined 4 dichotomized (yes/no) cardiometabolic risk factors: 1) elevated blood pressure (systolic value of ≥130 mm Hg or diastolic value of >85 mm Hg or ICD-9-CM code from 401.0 to 405.9); 2) elevated triglycerides (≥150 mg/dL or an ICD-9-CM code of 272.1); 3) low HDL cholesterol (<40 mg/dL for men and <50 mg/dL for women or an ICD-9-CM code of 272.5); and 4) prediabetes (HbA_1c_ value of 5.7%–6.4% or a fasting glucose value of 100–125 mg/dL or an ICD-9-CM code of 790.2x). Individuals were required to have either a fasting glucose test (84.7% of the total) or an HbA_1c_ test (40.4%).

b Data source: PORTAL weight cohort (7).

c All values are column percentages.

d All between-group comparisons are significant at *P* < .001 (1-way analysis of variance) because of the large sample size.

e Overweight defined as BMI of 23.0–27.4 for Asians and 25.0–29.9 for all other race/ethnicities.

f Class 1 obesity defined as BMI of 27.5–34.9 for Asians and 30.0–34.9 for all other race/ethnicities.

Compared with overweight, obesity Class I was associated with a 90% greater probability (OR, 1.90; 95% confidence interval [CI], 1.88–1.91) of having at least 1 CRF; Class II was associated with a nearly 3-fold greater probability (OR, 2.83; 95% CI, 2.80–2.87); and Class III was associated with a 4-fold greater probability (OR, 4.07; 95% CI, 4.00–4.14) (Figure). Older age was associated with a greater probability of having a CRF: by age 65, the probability of having at least 1 CRF was 5-fold greater (OR, 5.07, 95% CI, 5.00–5.14) than it was at age 20 to 34. Men were 45% more likely than women to have at least 1 CRF. Compared with non-Hispanic white adults, Hispanic, Asian, and Hawaiian/Pacific Islander adults were significantly more likely to have at least 1 CRF, and non-Hispanic black adults were 28% less likely.

**Figure:**
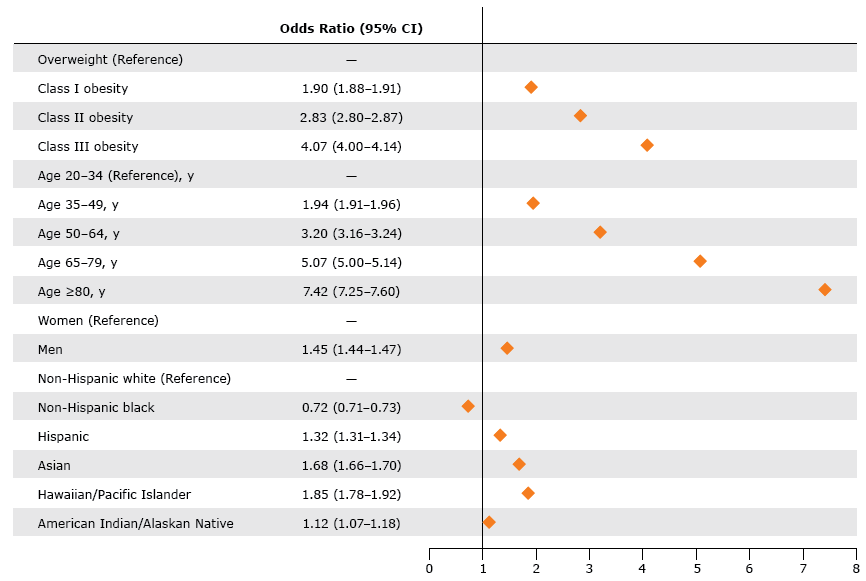
Multivariable adjusted probability of having 1 or more cardiometabolic risk factors among adults (aged ≥20 y) with overweight or obesity but not diabetes, Patient Outcomes Research to Advance Learning (PORTAL), 2012-2013. Because of the large sample size, 95% confidence intervals were narrow and cannot be depicted in the graphic. The logistic model equation was *Y* = −1.44 + 0.66 (Age Category 1) + 1.16 (Age Category 2) + 1.62 (Age Category 3) + 2.00 (Age Category 4) + 0.37 (Male) + 0.52 (Asian) – 0.33 (non-Hispanic black) + 0.28 (Hispanic) + 0.12 (American Indian/Alaskan Native) + 0.61 (Hawaiian/Pacific Islander) + 1.04 (Weight Category 1) + 1.40 (Weight Category 2) + 0.64 (Weight Category 3). The probability calculates as Probability(Outcome) = [exp(*Y*)]/(1+ exp(*Y*). **Table:** 

Variable	Odds Ratio (95% Confidence Interval)
**Weight**	
Overweight	1.00 [Reference]
Class I obesity	1.90 (1.88−1.91)
Class II obesity	2.83 (2.80−2.87)
Class III obesity	4.07 (4.00−4.14)
**Age, y**	
20–34	1.00 [Reference]
35–49	1.94 (1.91−1.96)
50–64	3.20 (3.16−3.24)
65–79	5.07 (5.00−5.14)
≥80	7.42 (7.25−7.60)
**Sex**	
Female	1.00 [Reference]
Male	1.45 (1.44−1.47)
**Race/ethnicity**	
Non-Hispanic white	1.00 [Reference]
Non-Hispanic black	0.72 (0.71−0.73)
Hispanic	1.32 (1.31−1.34)
Asian	1.68 (1.66−1.70)
Hawaiian/Pacific Islander	1.85 (1.78−1.92)
American Indian/Alaskan Native	1.12 (1.07−1.18)

Adults with complete clinical data were older but were otherwise similar to those with incomplete data; we found nearly identical distributions of overweight or obesity classes. Results were almost the same as those of the primary analytic sample when we reanalyzed the data using the entire 3,434,066-person sample and assumed those with a missing CRF value did not have that risk factor. The prevalence of each CRF was lower, but the odds ratios in the logistic regression were similar and the patterns were consistent with those of the primary analytic sample.

## Discussion

In this observational cohort study of approximately 1.3 million adults with overweight or obesity, we found that 4 commonly measured CRFs often associated with excess weight were prevalent. The prevalence of elevated blood pressure, elevated triglycerides, low HDL cholesterol, or prediabetes varied overall from 32% to 60% and, depending on the category of overweight or obesity, from 27% to 72%. However, 14% of the cohort had none of the 4 CRFs. Among adults with morbid obesity, 6% had none and 23% had only 1 CRF. Thus, although overweight or obesity is itself a CRF, the prevalence and number of the 4 CRFs of interest varied. 

The prevalence of CRFs varied across age groups; increased age was strongly and independently associated with CRFs. One interpretation of this finding is that young adults had not had sufficient time to develop risk factors. BMI is an imperfect measure of obesity for any age group and may inaccurately indicate the effects of obesity on adults older than 65 (10). Thus, we suggest caution when interpreting the associations of age-related CRFs and BMI on adults older than 65. However, in our study, adults in the 3 obesity classes were on average 3 years younger than adults in the overweight category, and adults with morbid obesity were on average 7 years younger than adults with overweight. Additionally, low HDL cholesterol was the most common CRF among adults younger than 35. Although our findings on age and HDL cholesterol may seem counterintuitive, they may have resulted from the study design, which excluded adults who had diabetes. If so, our results reflect the distribution of adults with overweight or obesity who are at risk for diabetes more accurately than previous reports. Our results underscore the concern that increases in the prevalence of obesity at ever younger ages is perhaps the most important public health problem in the United States. Low HDL cholesterol among young adults may be an early warning sign of CVD.

We limited our data to adults who had contact with the health system: each cohort member had at least 1 primary care visit recorded in the EHR. As a result, our cohort may not have included healthy adults who perceived no need for medical care. This potential bias was somewhat mitigated by our limiting the cohort to adults with overweight or obesity. Furthermore, even among adults with morbid obesity, some had none of the 4 CRFs. To the extent that healthy adults were excluded, we may have understated the proportion with none of the CRFs, especially among relatively young adults and those with low excess weight.

Individuals with obesity but no cardiometabolic abnormalities have been considered to have “metabolically healthy obesity” (MHO) (11), but the meaningfulness of this term is controversial. Individuals with MHO are at increased risk of type 2 diabetes (12–14), and a recent study showed that the obese phenotype, regardless of metabolic abnormalities, increases the risk of chronic kidney disease (15). MHO, however defined, is not a harmless condition, and may simply be evidence of slower metabolic responses to obesity that will equalize in the long run. Nevertheless, the risk of CVD and death among individuals with MHO is not clear. In a study using data from the third NHANES, rates of mortality risk among participants with obesity and cardiometabolic abnormalities and participants with MHO were similar and were nearly 3 times greater than rates for cardiometabolically normal participants with normal weight (16). Conversely, a clinical study of nearly 15,000 cardiometabolically healthy participants found that levels of coronary artery calcium were significantly greater among participants with obesity than among normal-weight participants, but the association was no longer significant after adjustment for CRFs (17). An observational study of more than 22,000 participants found that participants with MHO were not at higher risk of all-cause mortality or CVD (18). A recent systematic review reported that a minority of studies found associations between MHO and CVD and mortality, although 4 of 6 studies that examined subclinical CVD reported greater risk for patients with MHO than for cardiometabolically healthy patients with normal weight (19). Lack of uniformity in the definition of MHO is likely contributing to these conflicting results.

Given that nearly 35% of the US adult population has a BMI that indicates obesity (1), the variation in the number and type of CRFs that we report suggests the need for further research to clearly define the phenotypes of body weight to better identify risk and deliver appropriate interventions. Development of risk factors is a dynamic process: with each additional CRF, CVD risk rises exponentially (20). However, phenotypes may vary by CVD risk, which in turn depends on which CRFs are present and the order in which they develop (20). Study of the progression of cardiometabolic risk is a vitally important area for further research and requires a large data set with several years of longitudinal data. Our study’s observational design and cross-sectional data did not allow us to assess morbidity or mortality risk over time, but several more years of data have now accumulated at each participating site. Because PORTAL is just one of 11 CDRNs, all of which were required to generate a weight cohort using a common data model, the possibility of conducting such a study that includes patients from all 50 states is a reality (6).

The size of our cohort allowed us to explore differences in the prevalence of CRFs across classes of obesity among a diverse group of adults without diabetes. To our knowledge, such data have not been reported until now. Compared with non-Hispanic white adults, we found that Hispanic, Asian, and Hawaiian/Pacific Islander adults were substantially more likely to have CRFs, but non-Hispanic black adults were less likely. Although this finding may seem counterintuitive, it is consistent with a previous report (4). Furthermore, as with younger age, non-Hispanic black individuals with greater levels of obesity may have greater mortality and higher diabetes incidence rates than individuals in other racial/ethnic groups, factors that would disproportionately exclude them from our analytic data set. Our findings within and between racial/ethnic categories demonstrated disparities in the association between overweight or obesity and CRFs. Research is needed to determine whether current cut-points for assessing CRFs are appropriate for all races, ethnicities, and age groups and to determine how the presence or absence of CRFs affects cardiometabolic outcomes differently.

Our study has several limitations in addition to those mentioned previously. We were unable to include several other potential explanatory factors that differentiate patients within categories of overweight or obesity. First, the ratio of visceral fat to subcutaneous fat differentially affects a person’s risk of CVD and mortality (10,21), which is why most accepted definitions of metabolic syndrome require assessment of waist circumference rather than BMI (20). Second, cardiorespiratory fitness may play a more important role than body composition in developing CRFs, such that the “fat but fit” patient has less risk than the “lean but unfit” patient (22). The incidence of diabetes decreases as cardiorespiratory fitness increases, regardless of obesity (23). Third, in conjunction with exercise that produces cardiorespiratory fitness, diet can affect CRFs (24,25). Adults with overweight or obesity in our study who had no risk factors may have been actively attempting to lose weight through diet and exercise, thus producing a more favorable risk profile. Fourth, systemic inflammation, often measured by high-sensitivity C-reactive protein, contributes to cardiometabolic risk (26), although it is not commonly included in definitions of cardiometabolic health (14). Fifth, cigarette smoking independently affects CRFs and cardiometabolic events (24,25). Sixth, family history as a marker for genetic predisposition affects CRFs and cardiometabolic risk (27). Each of these 6 unmeasured variables, alone or in combination, could explain the differential distribution of the number of CRFs across overweight or obesity categories but were not available in our data set. However, the inclusion of these data as explanatory variables would not have changed the distributions of CRFs across the weight and demographic categories reported herein.

The comprehensiveness of the EHR data collected by the participating health systems fostered the assessment of cardiometabolic measurements among much of their patient populations. Nevertheless, a potential weakness of our study was that data for at least 1 of the CRFs studied were missing from the EHRs of more than half of the otherwise-eligible adults. The sensitivity analyses, which considered missing data as equivalent to not having the CRF, reduced the prevalence of CRFs, but the relationship between demographic characteristics and the probability of cardiometabolic health was not affected. This finding suggests our results are robust.

The size and diversity of our study population allowed us to examine overweight or obesity levels and classes of obesity in numbers sufficiently large to identify meaningful associations. However, the large sample size also resulted in statistically significant results even when differences were minute and clinically insignificant. In some of the participating health plans, individuals with low socioeconomic status may be underrepresented. However, all health plans (except one) included individuals covered by state-subsidized insurance, and Denver Health’s mission is to serve those with limited ability to pay for medical services. Nevertheless, our sample may not fully represent the unemployed and underemployed, all levels of education, and other social determinants of health. Adults with high incomes may also have been underrepresented. Although the PORTAL cohort is large, it does not include individuals from all 50 states and, therefore, may not be fully representative of the United States. Because adults with a BMI of less than 23.0 were not included in the data set, the overall effect of overweight or obesity on the prevalence of CRFs relative to normal weight cannot be evaluated. We could not ascertain whether triglyceride levels were measured in a fasting state or nonfasting state, which could have led to some misclassification of that CRF.

The number and type of adverse CRFs varied substantially among 1.3 million adults with overweight or obesity but not diabetes. Although most adults had at least 1 CRF, some had none. As recently recommended (20), future studies should examine the order in which CRFs develop in individuals with overweight or obesity and identify the characteristics of individuals who have none.

## References

[1] Ogden CL , Carroll MD , Kit BK , Flegal KM . Prevalence of childhood and adult obesity in the United States, 2011–2012. JAMA 2014;311(8):806–14. 10.1001/jama.2014.732 24570244 PMC4770258

[2] Geiss LS , Wang J , Cheng YJ , Thompson TJ , Barker L , Li Y , Prevalence and incidence trends for diagnosed diabetes among adults aged 20 to 79 years, United States, 1980–2012. JAMA 2014;312(12):1218–26. 10.1001/jama.2014.11494 25247518

[3] Gregg EW , Zhuo X , Cheng YJ , Albright AL , Narayan KMV , Thompson TJ . Trends in lifetime risk and years of life lost due to diabetes in the USA, 1985–2011: a modelling study. Lancet Diabetes Endocrinol 2014;2(11):867–74. 10.1016/S2213-8587(14)70161-5 25128274

[4] Wildman RP , Muntner P , Reynolds K , McGinn AP , Rajpathak S , Wylie-Rosett J , The obese without cardiometabolic risk factor clustering and the normal weight with cardiometabolic risk factor clustering: prevalence and correlates of 2 phenotypes among the US population (NHANES 1999–2004). Arch Intern Med 2008;168(15):1617–24. 10.1001/archinte.168.15.1617 18695075

[5] McGlynn EA , Lieu TA , Durham ML , Bauck A , Laws R , Go AS , Developing a data infrastructure for a learning health system: the PORTAL network. J Am Med Inform Assoc 2014;21(4):596–601. 10.1136/amiajnl-2014-002746 24821738 PMC4078291

[6] Fleurence RL , Curtis LH , Califf RM , Platt R , Selby JV , Brown JS . Launching PCORnet, a national patient-centered clinical research network. J Am Med Inform Assoc 2014;21(4):578–82. 10.1136/amiajnl-2014-002747 24821743 PMC4078292

[7] Young DR , Waitzfelder BA , Arterburn D , Nichols GA , Ferrara A , Koebnick C , The Patient Outcomes Research to Advance Learning (PORTAL) network adult overweight and obesity cohort: development and description. JMIR Res Protoc 2016;5(2):e87. 10.2196/resprot.5589 27307352 PMC4927804

[8] Nichols GA , Schroeder EB , Karter AJ , Gregg EW , Desai J , Lawrence JM , Trends in diabetes incidence among 7 million insured adults, 2006–2011: the SUPREME-DM project. Am J Epidemiol 2015;181(1):32–9. 10.1093/aje/kwu255 25515167 PMC4288120

[9] WHO Expert Consultation. Appropriate body-mass index for Asian populations and its implications for policy and intervention strategies. Lancet 2004;363(9403):157–63. 10.1016/S0140-6736(03)15268-3 14726171

[10] Winter JE , MacInnis RJ , Wattanapenpaiboon N , Nowson CA . BMI and all-cause mortality in older adults: a meta-analysis. Am J Clin Nutr 2014;99(4):875–90. 10.3945/ajcn.113.068122 24452240

[11] Després JP . What is “metabolically healthy obesity”?: from epidemiology to pathophysiological insights. J Clin Endocrinol Metab 2012;97(7):2283–5. 10.1210/jc.2012-2081 22774209

[12] Bell JA , Kivimaki M , Hamer M . Metabolically healthy obesity and risk of incident type 2 diabetes: a meta-analysis of prospective cohort studies. Obes Rev 2014;15(6):504–15. 10.1111/obr.12157 24661566 PMC4309497

[13] Nichols GA , Moler EJ . Diabetes incidence for all possible combinations of metabolic syndrome components. Diabetes Res Clin Pract 2010;90(1):115–21. 10.1016/j.diabres.2010.06.011 20630609

[14] Lotta LA , Abbasi A , Sharp SJ , Sahlqvist AS , Waterworth D , Brosnan JM , Definitions of metabolic health and risk of future type 2 diabetes in BMI categories: a systematic review and network meta-analysis. Diabetes Care 2015;38(11):2177–87. 10.2337/dc15-1218 26494809 PMC4826609

[15] Chang Y , Ryu S , Choi Y , Zhang Y , Cho J , Kwon M-J , Metabolically healthy obesity and development of chronic kidney disease: a cohort study. Ann Intern Med 2016;164(5):305–12. 10.7326/M15-1323 26857595

[16] Kuk JL , Ardern CI . Are metabolically normal but obese individuals at lower risk for all-cause mortality? Diabetes Care 2009;32(12):2297–9. 10.2337/dc09-0574 19729521 PMC2782994

[17] Chang Y , Kim BK , Yun KE , Cho J , Zhang Y , Rampal S , Metabolically-healthy obesity and coronary artery calcification. J Am Coll Cardiol 2014;63(24):2679–86. 10.1016/j.jacc.2014.03.042 24794119

[18] Hamer M , Stamatakis E . Metabolically healthy obesity and risk of all-cause and cardiovascular disease mortality. J Clin Endocrinol Metab 2012;97(7):2482–8. 10.1210/jc.2011-3475 22508708 PMC3387408

[19] Roberson LL , Aneni EC , Maziak W , Agatston A , Feldman T , Rouseff M , Beyond BMI: the “metabolically healthy obese” phenotype & its association with clinical/subclinical cardiovascular disease and all-cause mortality — a systematic review. BMC Public Health 2014;14:14. 10.1186/1471-2458-14-14 24400816 PMC3890499

[20] Sperling LS , Mechanick JI , Neeland IJ , Herrick CJ , Després JP , Ndumele CE , The CardioMetabolic Health Alliance: working toward a new care model for the metabolic syndrome. J Am Coll Cardiol 2015;66(9):1050–67. 10.1016/j.jacc.2015.06.1328 26314534

[21] Hamdy O , Porramatikul S , Al-Ozairi E . Metabolic obesity: the paradox between visceral and subcutaneous fat. Curr Diabetes Rev 2006;2(4):367–73. 10.2174/1573399810602040367 18220642

[22] Arsenault BJ , Lachance D , Lemieux I , Alméras N , Tremblay A , Bouchard C , Visceral adipose tissue accumulation, cardiorespiratory fitness, and features of the metabolic syndrome. Arch Intern Med 2007;167(14):1518–25. 10.1001/archinte.167.14.1518 17646606

[23] Juraschek SP , Blaha MJ , Blumenthal RS , Brawner C , Qureshi W , Keteyian SJ , Cardiorespiratory fitness and incident diabetes: the FIT (Henry Ford ExercIse Testing) project. Diabetes Care 2015;38(6):1075–81. 10.2337/dc14-2714 25765356

[24] Mozaffarian D , Benjamin EJ , Go AS , Arnett DK , Blaha MJ , Cushman M , Heart disease and stroke statistics — 2016 update: a report from the American Heart Association. Circulation 2016;133(4):e38–360. 10.1161/CIR.0000000000000350 26673558

[25] Daviglus ML , Lloyd-Jones DM , Pirzada A . Preventing cardiovascular disease in the 21st century: therapeutic and preventive implications of current evidence. Am J Cardiovasc Drugs 2006;6(2):87–101. 10.2165/00129784-200606020-00003 16555862

[26] Ridker PM , Hennekens CH , Buring JE , Rifai N . C-reactive protein and other markers of inflammation in the prediction of cardiovascular disease in women. N Engl J Med 2000;342(12):836–43. 10.1056/NEJM200003233421202 10733371

[27] Florido R , Zhao D , Ndumele CE , Lutsey PL , McEvoy JW , Windham BG , Physical activity, parental history of premature coronary heart disease, and incident atherosclerotic cardiovascular disease in the Atherosclerosis Risk in Communities (ARIC) study. J Am Heart Assoc 2016;5(9):e003505. 10.1161/JAHA.116.003505 27577582 PMC5079018

